# Postgraduate Courses in Pharmaceutical Medicine in Italy

**DOI:** 10.3389/fmed.2017.00079

**Published:** 2017-06-16

**Authors:** Domenico Criscuolo

**Affiliations:** ^1^Genovax, Colleretto Giacosa, Italy

**Keywords:** postgraduate education, Italy, pharmaceutical medicine, clinical trials, drug development

## Abstract

Italy has a significant tradition of excellence in the area of clinical trials (CTRs): important achievements in the clinical development of rifampicin and adriamycin, the two most famous drugs discovered in the research laboratories of two Italian pharmaceutical companies, paved the way to the establishment of a culture of clinical development, mainly in the areas of antimicrobials and oncology. Despite the fact that now the Italian market of pharmaceuticals is largely dominated by multinational companies with headquarters outside Italy, the contribution of Italian studies to the clinical development of new drugs is still significant. Indeed, it largely exceeds the percentage of Italian inhabitants versus the ones living in the remaining EU countries, as Italy has about 12% of EU population, but has a 17% share of the EU CTRs. Education in Pharmaceutical Medicine is now a must for all professionals interested to work either in pharma companies or in contract research organizations: several Italian universities are offering high quality courses, and in the last 10 years, more than 1,200 professionals received a postgraduate education in pharmaceutical medicine. This result places Italy on top of countries concerned about the professional education of people involved in drug development and will represent an asset for a larger involvement of Italian clinical sites in the global process of clinical research.

## Introduction

**Figure 1:**
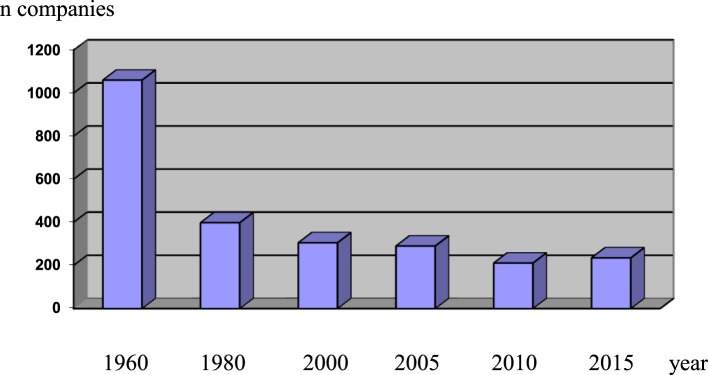
Number of pharmaceutical companies active in Italy from 1960 to 2015 (from Farmindustria).

The Italian pharmaceutical industry had a boom in the post World War 2 era (1), boosted by the desire for a better control of diseases and also by the lack of a law about patent protection (Figure 1). In this dynamic scenario, Italian chemists and pharmacologists had the opportunity to establish various research groups, and some of them achieved very important results. In particular, the research groups in Lepetit and in Farmitalia, two large companies based in the area of Milan, discovered rifampin and adriamycin. The clinical development of these two key medicines, always present in the WHO list of essential drugs, created a scientific and managerial environment, which formed the basis of a reputation of excellence of the Italian clinical research (2, 3).

In the following decades, the lack of political support and also a short vision of Italian industrial families stimulated several foreign multinational companies to acquire Italian pharmaceutical companies: from 1980 to 2000, the scenario drastically changed, with foreign multinational companies conquering about 80% of the Italian market (Figure 1). However, the positive image of excellence in clinical research was safeguarded, and Italian professionals continued to keep in good shape the value of clinical studies performed in Italian trial sites.

Indeed, the number of clinical trials (CTRs) performed in Italy from the year 2000 onward, recorded in a dedicated page of the website of the Italian Medicines Agency (AIFA) (4), moved from 557 to a peak of 880 in the year 2008: since then, the global crisis affecting the number of clinical studies had some consequences also in Italy, which, however, continues to keep a good number of CTRs (Table 1). The comparison with the other countries of the European Union is even more encouraging: Italy has always got a share of the total number of CTRs ranging from 16.4% (year 2011) to 18.2% (year 2014), and this share is stable above 17% (Table 2). This is a significant result, having in mind that the Italian population is made of about 60.5 million inhabitants and represents approximately 11.8% of the population of the 28 countries of the European Union, which in the year 2015 was about 510 million people. Of course, this is a generic indicator, which, however, is suggestive of an interesting trend.

**Table 1:** Number of clinical trials (CTRs) approved in Italy from 2000 to 2015.

Year	Number of approved CTRs
2000	557
2001	605
2002	560
2003	568
2004	624
2005	664
2006	778
2007	796
2008	880
2009	761
2010	670
2011	676
2012	697
2013	583
2014	592
2015	672

**Table 2:** Number of clinical trials approved in EU countries and in Italy from 2011 to 2015.

Year	*n* trials approved in EU countries	*n* trials approved in Italy	Percentage Italy/EU
2011	4,127	676	16.4
2012	3,943	697	17.7
2013	3,383	583	17.2
2014	3,249	592	18.2
2015	3,918	672	17.2

Quality in clinical research is a must: this process, which started in the 1980s with the implementation of the Good Clinical Practice methodology, is being continuously updated with revisions and amendments. Education in Pharmaceutical Medicine started to get interest from professionals in clinical research, who identified the need for dedicated university master courses devoted to all areas of medicines development: and the first master courses were established in Europe and in Italy at the end of the 1980s. Initially, these courses were attended by a minority of professionals, only stimulated by the personal wish to get a more comprehensive education in Pharmaceutical Medicine.

As Italy is concerned, the turning point of students’ participation to master courses in Pharmaceutical Medicine was in the year 2008. The Italian Medicines Agency, having in mind the relevant number of CTRs performed in the country (Table 1), issued a decree entitled “Definition of the minimum requirements, which Contract Research Organizations (CROs) shall satisfy in order to work within CTRs on medicinal products” (5). This decree imposed a significant mandatory training for all professionals wishing to work as clinical monitors and related tasks, lasting for 6 months: however, this training could be avoided in the event the clinical monitor had got a University master title in Pharmaceutical Medicine. This law was a tremendous boost to master courses: in fact, from the year 2008 onward, the majority of pharmaceutical companies and practically all CROs, whenever looking for new hires, require the master title, which will guarantee to have not only well trained professionals but also people ready to be active in their jobs. And in fact not only University masters established before the year 2008 had a significant increase in applications but several new master courses were established all over Italy, in order to satisfy an increasing demand of postgraduate courses in Pharmaceutical Medicine (Table 3).

**Table 3:** Master courses in pharmaceutical medicine and related areas, active in Italy, and number of professionals who got the master title.

^a^Catholic University—Rome (PharmaTrain Center of Excellence)	*n* = 300
^a^Bicocca University—Milano (PharmaTrain Center of Excellence)	*n* = 200
^a^State University “La Sapienza”—Rome	*n* = 30
^a^State University—Pisa	*n* = 40
^a^State University—Milano	*n* = 120
^a^State University “Tor Vergata”—Rome	*n* = 30
^a^State University—Florence	*n* = 150
^a^State University—Camerino	*n* = 30
^b^Second University—Naples	*n* = 80
^b^State University—Pavia	*n* = 100
^b^State University—Catania	*n* = 50
^b^State University—Verona	*n* = 40
^c^State University—Novara	*n* = 150

	Total *n* ≈ 1,320

*Learning outcomes are related to the following*.

*^a^Pharmaceutical medicine*.

*^b^Drug safety*.

*^c^Market access*.

Some years ago, in an evaluation of Pharmaceutical Medicine in Italy (6), I estimated that about 3,000 professionals were working in Italy on jobs closely related to all areas of medicines research and development: I presume that this number has now increased, mainly driven by the large number of new hires from CROs, possibly reaching the level of 5,000 people. It is noteworthy to underline that we have today in Italy about 25% of professionals devoted to drug development who have a postgraduate education in Pharmaceutical Medicine. It is my solid opinion that Italy is now the country with the largest number of professionals in clinical research with a postgraduate title: this result will certainly improve the quality of clinical studies performed in Italy, and possibly attract an even larger number of international trials.

Finally, let me add that the objective for a better quality is being continuously upgraded. So, what is the current scenario offering? The new target is the title of Specialist in Medicines Development (SMD), a joint program implemented by the PharmaTrain Federation (PTF) (7) and the International Federation of Associations of Pharmaceutical Physicians and Pharmaceutical Medicine (IFAPP) (8). These two federations have collaborated since the year 2009 in the IMI funded program called “PharmaTrain” (9), which aimed at harmonizing the programs of European master courses in Pharmaceutical Medicine. The Pharmatrain program achieved a good success, and from 2009 to 2014 was able to create a network of 15 master courses run in European countries and adopting the same syllabus. This innovative idea of harmonization is now further supported by the PTF, which has in addition launched the SMD project. This program is being offered to all professionals with a master title, and is a 2-year supervision on the job, managed by a tutor, who is responsible to make sure that the applicant becomes expert in the seven domains of Pharmaceutical Medicine (10).

## Conclusion

Pharmaceutical Medicine is a profession, which requires a regular update of competencies, as drug development opens continuously new frontiers: postgraduate master courses are now widely available, with a harmonized syllabus, which guarantees a similar education on a global basis. The SMD program, which is being initially implemented in Italy and in Japan, starting in 2017, represents an additional step of the quality ladder: a must for all professionals in Pharmaceutical Medicine.

## Author Contributions

The author confirms being the sole contributor of this work and approved it for publication.

## Conflict of Interest Statement

The author declares that the research was conducted in the absence of any commercial or financial relationships that could be construed as a potential conflict of interest. The reviewer, DD, and handling editor declared a past co-authorship with the author, but the handling editor ensured that the process met the standards of a fair and objective review.
